#  Low-dose radiation risk and individual variation in radiation sensitivity in Fukushima

**DOI:** 10.1093/jrr/rrv053

**Published:** 2015-09-07

**Authors:** Hisanori Fukunaga, Akinari Yokoya

**Affiliations:** 1 Soma General Hospital, 142 Tsubogasaku, Niinuma, Soma, Fukushima 976-0011, Japan; 2 Research Group for Radiation and Biomolecular Science, Quantum Beam Science Center, Japan Atomic Energy Agency, 2-4 Shirakata-Shirane, Tokai, Ibaraki 319-1195, Japan

**Keywords:** Fukushima Daiichi nuclear accident, radiation exposure monitoring, low-dose radiation risk, radiation sensitivity

Dear Editor,

Although it has been gradually recognized that a range of low-dose (or low-dose-rate) radiation effects on living cells are possible key factors in evaluating ‘low-dose radiation risk’, there remains little of the coherence required among robust data that can be used with confidence in risk assessments [1]. It is true that carcinogen risk should be evaluated carefully by considering both clear dose–response relationships and accurate exposure measurements [2]. It may take some time to incorporate mechanistic aspects into risk assessments, and the individual-level accumulated radiation exposure dose has been the sole indicator in terms of radiation protection.

At present, residents of Fukushima, especially those in Hamadōri, the eastern part of Fukushima, continue to be gripped by fear of contamination by radioactive substances; however, regardless of difficulties, medical personnel continue to cultivate close relationships with the patients. From previous statistical data, the long-term risk of cancer from exposure to radiation clearly increases at doses exceeding 100 mSv; therefore, following Japan's worst-ever accident, at the Fukushima Daiichi nuclear power plant (FNPP), radiation doses have been measured among Fukushima residents through the use of both personal dosimeters (for external exposure) and whole-body counters (for internal exposure) (Fig. 1) [3].

**Fig. 1.**
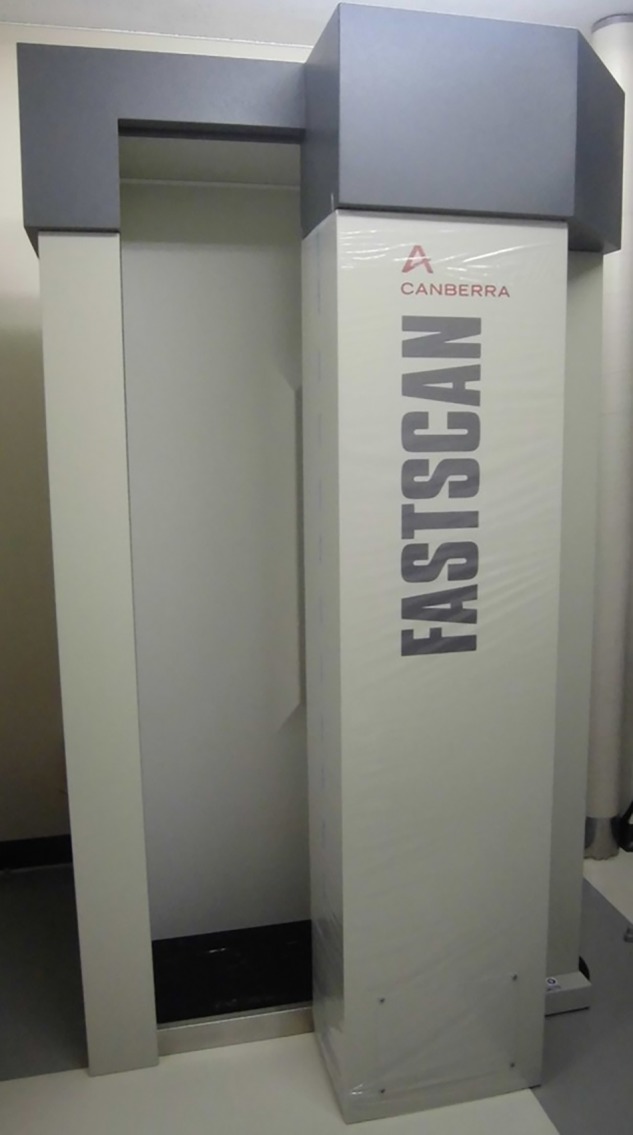
A whole-body counter (Canberra's Fastscan System) in Soma General Hospital. The use of this equipment has been one of the most common methods of detecting total internal radiation exposure following the 2011 nuclear crisis in Fukushima.

From the viewpoint of precision medicine [4], we note a possible issue: there might be Fukushima residents, including young children, with greater than average radiation sensitivity because of their genetic background. There is no evidence that the cancer risk of such residents exposed to <100 mSv is higher than that of ‘normal’ residents; however, the investigation of individual variation in radiation sensitivity in Fukushima seems to be insufficient. Radiation sensitivity substantially varies depending on individual genetic background, as suggested by the results of NASA's research into space radiation effects on astronauts [5]. Micronucleus frequency in lymphocytes is thought to reflect an increased cancer risk later in life [6]. A linear dose–response curve of micronucleus frequency is commonly observed in the dose range of 20–100 mGy, and doses in this range have been known to induce increased frequencies of micronuclei in cells from individuals with defective DNA damage response genes (e.g. *ATM* [ataxia telangiectasia mutated gene], *DCLRE1C* [DNA cross-link repair 1C gene] and *LIg4* [DNA ligase IV gene]) [7]. A number of adverse reactions to radiation therapy in individuals suffering from DNA damage response defective disorders, such as ataxia–telangiectasia (A–T), Nijmegen Breakage Syndrome, Fanconi anemia (FA) and DNA LIG4-deficiency, have been reported [8]. Patients with these disorders and heterozygous carriers as well can be associated with radiation sensitivity, as well as with cancer predisposition, although the underlying mechanism of radiation-induced carcinogenesis in this group remains to be determined [9]. As shown in Table 1, the estimated prevalence of heterozygous carriers for several DNA repair disorders in the general population has been reported [10–14]. According to statistical data from the Fukushima prefecture municipality, as of 1 April 2011 there were 2 014 603 Fukushima residents [15], suggesting that ∼20 000 heterozygous carriers of A–T were living in Fukushima immediately after the FNPP accident. It is likely that numerous genes, not only DNA damage response genes, potentially contribute to the regulation of radiation sensitivity in humans, and individual variations in radiation sensitivity could be much larger than expected. Although public health administrators should make concerted efforts to minimize the radiation-induced cancer risk of Fukushima residents, especially children, there appears to have been too little consideration of their genetic background and how it might affect their radiation sensitivity.

**Table 1.** Estimated prevalence of heterozygous careers of DNA repair disorders

DNA repair gene	Responsible disease	Estimated prevalence
*ATM*	Ataxia telangiectasia	1:100
*WRN*	Werner syndrome	1:100
*FANCA, FANCC, FANCG*	Fanconi anemia	1:181 (in the USA)
*MLH1, MSH2, MSH6, PMS2, EPCAM*	Lynch syndrome	1:370 (or higher)
*BRCA1, BRCA2*	Hereditary breast ovarian cancer syndrome	1:400–800

In conclusion, given the influence of precision medicine in this matter, we are concerned about the use of radiation exposure levels as the sole criterion in evaluating cancer risk among residents living near the FNPP accident site. The potential risk of radiation-induced cancer in Fukushima could be small at the population level due to the scarcity of patients and heterozygous carriers with radiosensitivity disorders; however, it should not be ignored [16]. We hereby propose that not only a radiation exposure assessment but also additional medical checkups for cancer, such as ultrasonography, gastrointestinal endoscopy, measurements of tumor markers in blood and urine, and genetic testing, should be combined in a balanced fashion to minimize the number of local residents who will develop advanced cancers in the future.

## FUNDING

Funding to pay the Open Access publication charges for this letter was provided by Hisanori Fukunaga.
